# Enhanced Ethanol Gas Sensing Properties of SnO_2_-Core/ZnO-Shell Nanostructures

**DOI:** 10.3390/s140814586

**Published:** 2014-07-21

**Authors:** T. Tharsika, A. S. M. A. Haseeb, Sheikh A. Akbar, Mohd Faizul Mohd Sabri, Wong Yew Hoong

**Affiliations:** 1 Department of Mechanical Engineering, Faculty of Engineering, University of Malaya, Kuala Lumpur 50603, Malaysia; E-Mails: tharsika@siswa.um.edu.my (T.T.); faizul@um.edu.my (M.F.M.S.); yhwong@um.edu.my (Y.H.W.); 2 Center for Industrial Sensors and Measurements (CISM), Department of Materials Science and Engineering, Ohio State University, 2041 College Road, Columbus, OH 43210, USA; E-Mail: akbar.1@osu.edu

**Keywords:** ethanol gas sensor, ZnO, SnO_2_, core-shell nanostructures, hierarchical nanostructures

## Abstract

An inexpensive single-step carbon-assisted thermal evaporation method for the growth of SnO_2_-core/ZnO-shell nanostructures is described, and the ethanol sensing properties are presented. The structure and phases of the grown nanostructures are investigated by field-emission scanning electron microscopy (FESEM), transmission electron microscopy (TEM) and X-ray diffraction (XRD) techniques. XRD analysis indicates that the core-shell nanostructures have good crystallinity. At a lower growth duration of 15 min, only SnO_2_ nanowires with a rectangular cross-section are observed, while the ZnO shell is observed when the growth time is increased to 30 min. Core-shell hierarchical nanostructures are present for a growth time exceeding 60 min. The growth mechanism for SnO_2_-core/ZnO-shell nanowires and hierarchical nanostructures are also discussed. The sensitivity of the synthesized SnO_2_-core/ZnO-shell nanostructures towards ethanol sensing is investigated. Results show that the SnO_2_-core/ZnO-shell nanostructures deposited at 90 min exhibit enhanced sensitivity to ethanol. The sensitivity of SnO_2_-core/ZnO-shell nanostructures towards 20 ppm ethanol gas at 400 °C is about ∼5-times that of SnO_2_ nanowires. This improvement in ethanol gas response is attributed to high active sensing sites and the synergistic effect of the encapsulation of SnO_2_ by ZnO nanostructures.

## 1. Introduction

Solid state gas sensors are widely used for detecting various concentrations of toxic and combustible gases [1–3]. These sensors can be categorized into three main groups according to their sensing mechanisms: solid electrolyte gas sensors, semiconductor gas sensors and catalytic combustion gas sensors [4]. Among these sensors, oxide-based semiconductor gas sensors play an important role in various applications, such as environmental monitoring, personal safety and the control of industrial processes [4,5]. ZnO, SnO_2_, WO_3_, In_2_O_3_ and TiO_2_ are widely studied oxides for gas sensing applications [6–8]. Various approaches have been used to improve the response and selectivity of these oxides [9]. These include the addition of noble metals [10–13], the doping of metal oxide catalyst [14,15], developing hybrid sensing materials consisting of binary or ternary phase metal oxide systems [16,17] and the development of nanostructures with different morphologies, a large surface area and a smaller size [18–21]. Among the approaches used, tailoring of oxide nanostructures presents a good potential for tuning the sensitivity and selectivity during gas sensing. The use of a core-shell structure has been of particular interest. To date, α-Fe_2_O_3_/SnO_2_ [22–24], GaN/WO_3_ [25], TiO_2_/ZnO [26,27], multi-walled CNT/SnO_2_ [28], ZnO/SnO_2_ [29–31], CeO_2_/TiO_2_ [32], Fe_2_O_3_/ZnO [33,34], MoO_3_/TiO_2_ [35] and Fe_2_O_3_/TiO_2_ [36] core-shell nanostructures have been investigated for gas sensing.

Important progress was made recently by Park *et al.* [26], who reported a five-fold increase in sensitivity for ethanol sensors based on TiO_2_/ZnO core-shell nanorods as compared with mere TiO_2_ nanorods. Chen *et al.* [28] reported that multi-walled CNT/SnO_2_ core/shell nanostructures showed enhanced sensitivity of ethanol up to 24.5 at 50 ppm. These results showed that enhanced sensing was due to the small size of SnO_2_ nanoparticles formed on top of multi-walled CNT and the special morphology of the core-shell heterostructure. Park *et al.* [31] demonstrated that SnO_2_-core/ZnO-shell nanowire sensors exhibited an especially increased response under UV illumination. The sensitivity of core-shell nanowire sensors towards 1–5 ppm of NO_2_ was improved by 2–3-times compared with pristine SnO_2_ or ZnO nanowires. Zhang *et al.* [34] reported that an α-Fe_2_O_3_/ZnO core-shell nanospindle sensor showed a sensitivity of 17.8 to an ethanol concentration of 100 ppm, which was more than two-times higher as compared with that of pristine α-Fe_2_O_3_ nanospindles sensor. The significant enhancement in the sensor response was attributed to its unique core-shell structure.

In this study, SnO_2_/ZnO core-shell-type nanowires and hierarchical nanostructures are prepared by a single-step carbon-assisted thermal evaporation method under ambient pressure. The fabrication of these nanostructures simply involves the evaporation of commercial metal oxide powders mixed with activated carbon at elevated temperatures in an inert gas atmosphere. In this process, all nanostructures are formed directly from the vapor phase in the absence of a metal catalyst, which is referred to as the vapor-solid (VS) growth [37]. In some cases, vacuum conditions [38] and strict temperature control [39] are necessary for the formation of nanowires in the vapor phase, because some materials may not sublimate under ambient pressure (normal atmosphere). An effective way to generate the necessary vapor phase under ambient pressure is to add activated carbon [40]. In our previous work [41], we reported the formation of SnO_2_-core/ZnO-shell nanowires at a growth time of 30 min and hierarchical SnO_2_-core/ZnO-shell nanostructures at a growth time of 120 min. In this paper, detailed structural characterizations of fabricated SnO_2_-core/ZnO-shell nanostructures are reported for various growth times. Gas sensors are fabricated using these nanostructures and tested for ethanol, hydrogen and methane gases.

## 2. Experimental Section

### 2.1. Preparation of SnO_2_-Core/ZnO-Shell Nanostructures

**Figure 1.**
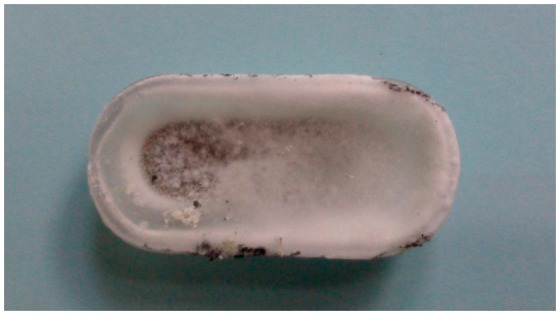
Nanostructures visible as a white fluffy mass with naked eyes formed at the edges and the top of the quartz boat.

SnO_2_-core/ZnO-shell nanostructures were synthesized on the top and the edges of a quartz boat by the single-step carbon-assisted thermal evaporation method, as reported earlier [41]. Zinc oxide, tin (IV) oxide and activated carbon powders at a ratio of 9:1:10 were loaded in a quartz boat after being mixed in a ball mill for 8 h. Then, the quartz boat was placed at the center of a horizontal tube furnace. The furnace was heated to 900 °C, and the temperature was maintained for different growth times of 15, 30, 60, 90 and 120 min, followed by cooling down to room temperature. A carrier gas of Ar (purity 99.99%) was flowed at a rate of 25 mL/min during the growth period. A white fluffy mass was formed on the top and the edges of the quartz boat, as shown in Figure 1.

### 2.2. Characterization

The white fluffy mass was collected from the quartz boat and spread on a carbon tape for characterization. The structure of the SnO_2_/ZnO nanostructures grown for different time periods was examined by X-ray diffraction (XRD) using a Siemens D-5000 model with a monochromatic CuK_α_ radiation (λ = 1.5406 Å) and Ni filter. The current and operating voltage were 40 mA and 40 kV, respectively. The scanning was performed between 25° to 70°, with a scanning step of 0.03°/s. Electron microscopy was used to investigate the structure and morphology of SnO_2_-core/ZnO-shell nanostructures. A morphological investigation of the fabricated nanostructures was carried out by Auriga Zeiss Ultra-60 field emission scanning electron microscopy (FESEM). The high magnification image and the elemental distribution in the SnO_2_/ZnO nanostructures were obtained by the transmission electron microscope (TEM, FEI Tecnai F-20 microscopy) equipped with an energy dispersive X-ray spectroscope. Samples for TEM imaging were prepared by scratching samples from the quartz boat and dispersed in deionized water followed by sonication for 5 min. A drop of the suspension was then dropped onto a carbon-coated Cu grid (300 mesh) using a micropipette.

### 2.3. Fabrication of Sensor and Measurements

Inks for fabricating the sensors were prepared by suspending the nanostructures as follows: Initially, 46 wt % of α-terpineol, 8 wt % of ethyl cellulose and 46 wt % of diethylene glycol dibutyl ether (DGDE) were weighed individually. Then, α-terpineol and DGDE were mixed at room temperature in a glass container. Ethyl cellulose was then added to the mixture and heated on a hotplate until it was completely dissolved. The synthesized nanostructures (1 mg) were added to the ink and ultrasonicated for 5 min. Finally, the ink was drop-coated on alumina substrate having dimensions of 5 mm × 5 mm with a printed Au electrode to form the gas sensor. After drying in air at room temperature, the as-prepared sensor device was heat treated at 700 °C for 2 h. The sensor was placed inside a horizontal tube furnace, and a sensing measurement was carried out using the desired test gas at various gas concentrations of 20, 50, 100, 250 and 400 ppm with N_2_ background. The sensor response was defined as the ratio of (R_a_−R_g_)/R_a_, where R_a_ and R_g_ are the resistances measured in nitrogen and tested gas, respectively. The response and recovery times are defined as the time taken by the sensor to attain 90% of the response and recovery signals, respectively [42].

## 3. Results and Discussion

Figure 1 shows the white fluffy mass that was deposited in the quartz boat during the growth of the nanostructure. In this process, the metal vapor is created by the reaction between zinc oxide, tin oxide and activated carbon. The vapor is transported by Ar carrier gas and condenses around the edges and the top of the boat. According to the Ellingham diagram [43], possible reactions involved in the growth process of nanostructures at the deposition temperature used in the present study are:

(1) $$ZnO_{\left(s\right)}/SnO_{2(s)}+C_{\left(s\right)}\to Zn_{\left(v\right)}/Sn_{\left(v\right)}+CO_{\left(v\right)}/CO_{2(v)}$$

(2) Sn+(v)Zn(v)+3CO(v)→SnO2(s)+ZnO(s)+3C(s)

### 3.1. Structural Analysis of Nanostructures

**Figure 2.**
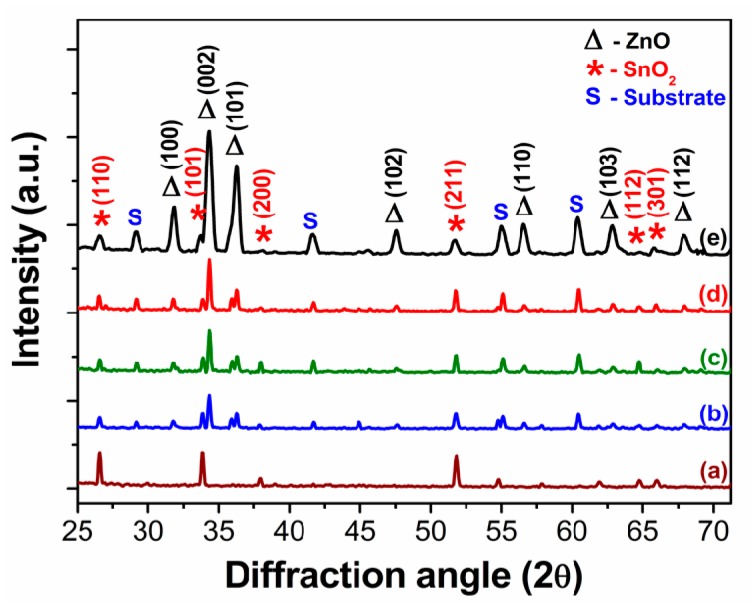
XRD patterns of SnO_2_/ZnO nanostructures obtained at different growth times: (**a**) 15 min; (**b**) 30 min; (**c**) 60 min; (**d**) 90 min and (**e**) 120 min.

XRD diffraction patterns of nanostructures (Figure 2) confirmed the crystal structure of the as-grown SnO_2_/ZnO nanostructures. XRD results show that SnO_2_ and ZnO nanostructures exhibit the tetragonal rutile structure and hexagonal wurtzite structure, respectively. These agree well with those in the standard powder diffraction files Nos. 88-0287 and 79-0208, respectively. Some peaks in XRD diffraction patterns are identified to be from the carbon substrate that was used to hold the nanostructures on the XRD stub. Pure SnO_2_ nanowires were identified only at a lower growth time of 15 min. With the increase of the growth time from 30 min to 120 min, the intensity of ZnO peaks increased, and the presence of the SnO_2_ phase was identified. Sharp diffraction peaks confirm that the phases possess good crystallinity.

**Figure 3.**
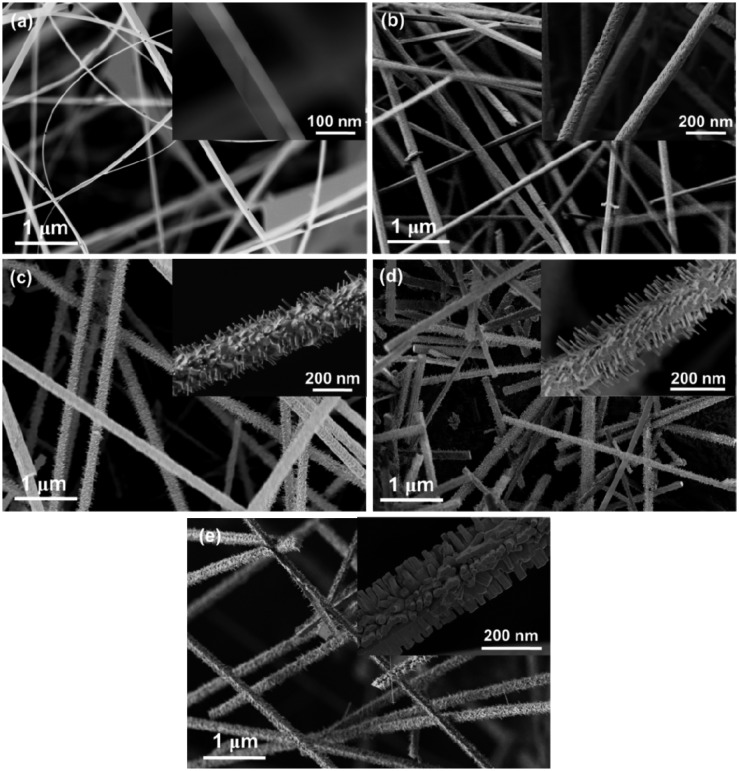
FESEM images of SnO_2_/ZnO nanostructures obtained at different growth times: (**a**) 15 min; (**b**) 30 min; (**c**) 60 min; (**d**) 90 min and (**e**) 120 min. The insets show details of a single nanowire.

Figure 3 shows FESEM images of SnO_2_/ZnO nanostructures grown for different growth times. Insets in the figures represent the magnified FESEM images of a single nanostructure. From Figure 3, it is obvious that the nanowires are transformed to a hierarchical nanostructure with increasing growth time. Rectangular cross-sectional nanowires are observed, when the growth time is 15 min (Figure 3a). Similar morphologies of SnO_2_ nanowires were previously reported by other authors [44–48]. The inset of Figure 3a exhibits the width-to-thickness aspect ratio of the nanowire to be around 2:1. Actually, the width ranges from 20 to 50 nm with an average length of ∼30 μm. The nanowires grown for 30 min have a circular cross-section with several tens of micrometers in length and a diameter ranging from 50 to 100 nm (Figure 3b). The inset of Figure 3b shows the magnified view of a circular nanowire with a diameter of ∼70 nm. With the increase of the growth time from 60 min to 120 min, the amount of hierarchical nanostructures increased (Figure 3c–e). At the same time, the length of the branch in hierarchical nanostructures increased from 50 nm to 150 nm, and the diameter also increased. For a long growth time of 120 min, all nanowires were completely changed to hierarchical nanostructures and individual branches merged into thicker branches (Figure 3e).

**Figure 4.**
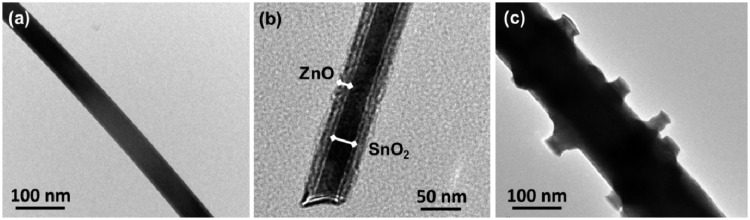
Low-magnification TEM images of a single nanostructure grown for different deposition time periods: (**a**) 15 min; (**b**) 30 min and (**c**) 120 min.

Figure 4 shows TEM images of SnO_2_/ZnO nanostructures grown for different time periods. The TEM image of a SnO_2_ nanowire grown for 15 min (Figure 4a) reveals that its average width is around 40 nm, and it is very uniform along its length. Figure 4b shows the TEM image of a typical SnO_2_-core/ZnO-shell nanowire obtained at a growth time of 30 min. The image shows that the diameter of the core is around 40 nm and the thickness of the shell layer is ∼20 nm. Catalyst tips are not observed, indicating that the growth of the nanostructures is not a vapor-liquid-solid (VLS), but a vapor-solid (VS) mechanism [49]. A low magnification TEM image of the hierarchical nanostructure obtained for a growth time of 120 min is shown in Figure 4c. The most part of the branches presumably broke off during TEM sample preparation by ultrasonication. It can be seen that the diameter of nanostructures increased with increasing deposition time.

**Figure 5.**
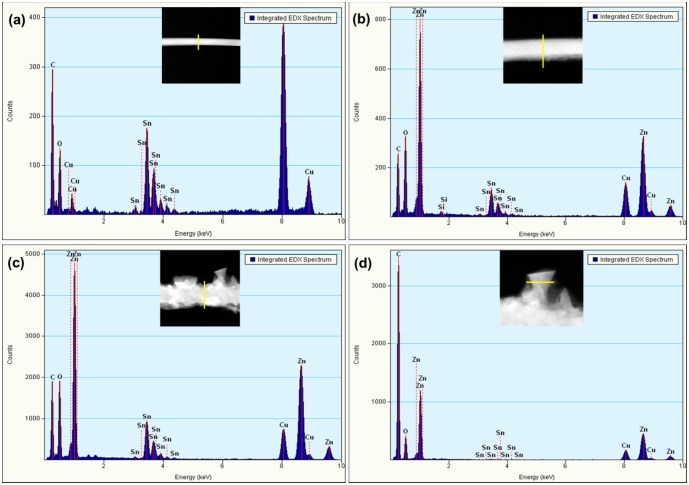
TEM integrated EDS line spectra of SnO_2_-core/ZnO-shell nanostructures grown for (**a**) 15 min; (**b**) 30 min; (**c**) 120 min (trunk of the nanostructure) and (**d**) 120 min (branch of the nanostructure).

Integrated EDS spectra collected along a line on the SnO_2_/ZnO nanostructures under the TEM at various growth times are shown in Figure 5a–d. It reveals that the intensity of Zn content increases with increasing growth time. Figure 5a shows the integrated line profile EDS spectrum obtained for the growth time of 15 min. It clearly exhibits that only pure SnO_2_ nanowires are observed. The EDS spectrum of SnO_2_-core/ZnO-shell nanowires grown for 30 min shown in Figure 5b exhibits that both Sn and Zn are seen in the nanostructure. Figure 5c,d shows the EDS spectra taken at the trunk and branch of the hierarchical nanostructure, which is obtained for the growth time of 120 min. As seen in Figure 5c, both Sn and Zn are detected in the trunk of the hierarchical nanostructures. However, only Zn and O are detected for the branch of the hierarchical nanostructure (Figure 5d). Therefore, we can conclude that all branches are made up of pure ZnO, which are grown on top of SnO_2_-core/ZnO-shell nanostructures.

### 3.2. Growth Mechanism of SnO_2_-Core/ZnO-Shell Nanostructures

At a lower growth time of 15 min, SnO_2_ nanowires with a rectangular cross-section were obtained (inset Figure 3a). Generally, a compound with a lower vapor pressure condenses easily to form a solid phase as compared with a compound possessing a higher vapor pressure. It is reported that SnO_2_ has a lower vapor pressure than ZnO at the deposition temperature of 900 °C [50]. Due to the lower pressure, SnO_2_ starts to nucleate first and forms the core of the nanowires at a shorter growth time of 15 min. If the growth time is further increased to 30 min, the partial pressure of SnO_2_ goes down, as the source (ZnO:SnO_2_ = 9:1) contains more ZnO than SnO_2_. This makes the vapor saturated in ZnO_(v)_ and creates a condition for ZnO to condense on top of the SnO_2_ nanowires in the form of a shell. Therefore, SnO_2_-core/ZnO-shell nanowires are observed at a deposition time of 30 min (inset of Figure 3b). Furthermore, hierarchical nanostructures are observed when the growth time further increases from 60 min to 120 min. We suggest that the growth of hierarchical nanostructures can be divided into two steps. In the first step, the SnO_2_ trunk nanowires grow, while the second step involves the growth of the ZnO shell layer on top of the SnO_2_ core. The thickness of the ZnO shell increases as more Zn vapor condenses with increased growth time. When the thickness of the ZnO shell layer reaches a critical value, then it acts as the seed layer for the growth of ZnO branches on top of the ZnO shell layer [51]. When the growth time increases to 120 min, SnO_2(v)_ is mostly consumed, as source mixture contains more ZnO_(s)_ than SnO_2(s)_. Therefore, ZnO hierarchical nanostructures can grow with a further increase of growth time; whereas for longer growth time of 120 min, nanorods branches tend to merge into wider structures. All branches exhibit a hexagonal cross-section, which consist of ZnO only (inset Figure 3e). These branches come out perpendicular to the trunk of nanowires. While, most of the hybrid nanostructures have four-fold symmetry branches, a few six-fold symmetry nanowires were also reported [52].

The morphology of the nanostructures depends on the growth parameters, including growth temperature, and the amount of starting materials [53]. Zhang *et al.* [53] found that a starting mixture of aluminum and alumina powder around 200 mg in weight favored the growth of regular Al_2_O_3_ nanowires, while a larger amount of starting material around 500 mg induced the hierarchical nanostructures. In our case, the amount of ZnO and SnO_2_ powder in the starting material is around 732 mg and 151 mg, respectively. A larger amount of ZnO in the starting mixture is another reason for the growth of ZnO nanorod branches at a longer deposition time (120 min).

### 3.3. Gas Sensing Properties of SnO_2_-Core/ZnO-Shell Nanostructures

**Figure 6.**
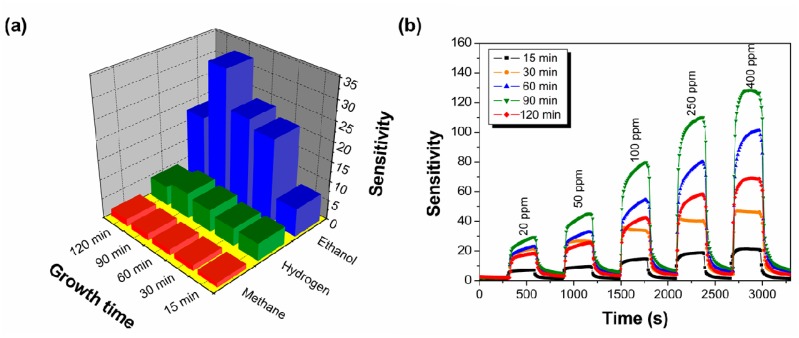
(**a**) Selectivity bar chart of sensors for response towards 20 ppm of methane, hydrogen and ethanol at 400 °C for different growth times of SnO_2_-core/ZnO-shell nanostructures; (**b**) Five cycles of response-recovery characteristics of various growth times of SnO_2_-core/ZnO-shell nanostructure sensors exposed to different ethanol concentrations.

The bar chart of sensor responses of SnO_2_-core/ZnO-shell nanostructures in terms of sensitivity towards various gases at a fixed concentration of 20 ppm and a sensing temperature of 400 °C is illustrated in Figure 6a. It is noteworthy that SnO_2_-core/ZnO-shell nanostructures exhibit higher sensitivity towards ethanol than just SnO_2_ nanowires (growth time of 15 min). As shown in Figure 6, SnO_2_-core/ZnO-shell nanostructures grown for 90 min have the highest response towards ethanol. It is also seen that the sensors exhibit a poor response to hydrogen and are almost insensitive to methane. Selectivity coefficients are calculated between the highest response of the test gas and the other test gases. The selectivity coefficients of K_ethanol/hydrogen_ and K_ethanol/methane_ (where, K_ethanol/hydrogen_ and K_ethanol/methane_ are the sensitivity ratio between ethanol and hydrogen, and ethanol and methane, respectively) for SnO_2_-core/ZnO-shell nanostructures grown for 90 min are determined to be around eight and 32, respectively, to 20 ppm of test gases. The results indicate that the fabricated SnO_2_-core/ZnO-shell nanostructures grown for 90 min have a much higher selectivity toward ethanol. Liu *et al.* [54] reported that a sensor prepared using a 3D hierarchical porous ZnO structure functionalized by Au nanoparticles exhibited a sensitivity of 8.9 towards 50 ppm ethanol. Xue *et al.* [55] demonstrated that a Pt-loaded SnO_2_ nanorod sensor showed a sensitivity up to 9.5 towards 50 ppm of ethanol. Compared with the reported results, our results show that high sensitivity and selectivity towards ethanol sensors can be achieved without additives.

Figure 6b represents the dynamic gas response of the SnO_2_-core/ZnO-shell nanostructures to various ethanol concentrations of 20, 50, 100, 250 and 400 ppm at 400 °C. Obviously, SnO_2_-core/ZnO-shell hierarchical nanostructures exhibit higher sensitivity to ethanol than just SnO_2_ nanowires, which is obtained for a deposition time of 15 min. The enhancement of the ethanol gas sensing performance of hierarchical SnO_2_-core/ZnO-shell nanostructures can be attributed to the greater sensing surface area due to the hierarchical structure. It can be seen from Figure 6b that when exposed to 20 ppm ethanol, the responses are about 6.1, 20.4, 22.5, 31.9 and 17.3 for SnO_2_/ZnO nanostructures grown for 15, 30, 60, 90 and 120 min, respectively. The results show that optimum sensing was observed for the nanostructures grown for 90 min. As the ethanol concentration increased from 20 ppm to 400 ppm, the sensitivity increased from 32.9 to 128. Therefore, nanostructures grown for 90 min exhibit nearly a five-fold enhanced sensitivity compared to just SnO_2_ nanowires (which was obtained for a growth time of 15 min). A sudden drop in the ethanol response was observed when the nanostructures were grown for 120 min. A possible explanation for this is given later in the discussion of the mechanism.

### 3.4. Gas Sensing Mechanism of SnO_2_-Core/ZnO-Shell Nanostructures

**Figure 7.**
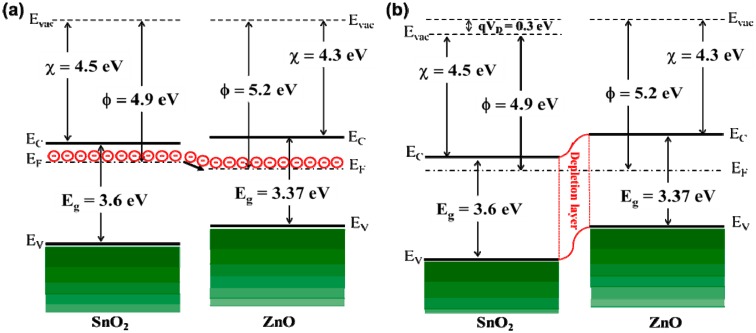
Energy band diagram of (**a**) SnO_2_ and ZnO and (**b**) SnO_2_-core/ZnO-shell nanostructures.

According to the above sensing results, SnO_2_-core/ZnO-shell nanostructure sensors exhibit significantly improved ethanol sensing properties compared with just SnO_2_ nanowires grown for 15 min. The improvement in the sensing property is thought to be related to the core/shell nanostructures. ZnO and SnO_2_ are n-type semiconductors with different band gaps, electron affinities and work functions. The electron affinity (*χ*), work function (*φ*) and band gap (*E_g_*) of ZnO are 4.3 eV, 5.2 eV and 3.37 eV, respectively, while for SnO_2_, these are 4.5 eV, 4.9 eV and 3.6 eV, respectively [56–58]. As seen in Figure 4b, a heterojunction has formed at the interface between SnO_2_-core and ZnO-shell. Figure 7 shows the possible energy band diagram of the fabricated SnO_2_-core/ZnO-shell nanostructures. Figure 7a shows that ZnO has a lower work function than SnO_2_. Therefore, electrons are transferred from SnO_2_ to ZnO until both Fermi levels become equal [56], which is shown in Figure 7b. An electron depletion layer is formed at the interface between SnO_2_ and ZnO, leading to a heterojunction barrier due to band bending. The enhanced sensing properties is ascribed to the variation of the heterojunction barrier when exposed to various kinds of gases [32].

Electron depletion region theory is widely used for sensing mechanism [59]. Electrons in the ZnO shell is depleted by adsorbing oxygen molecules from the air, leading to the formation of various ionized forms (O_2_^−^, O^2−^, O^−^) [60] when the SnO_2_-core/ZnO-shell nanostructures are exposed to air. When the core/shell sensor is exposed to ethanol, it reacts with adsorbed oxygen and releases the trapped electrons back to the conduction band of the ZnO shell by the dehydrogenation process [36], because ZnO and SnO_2_ are basic oxides. Thus, the resistance of the core/shell sensor is decreased, and the response is augmented. This fact is also supported by the fact that the resistance of just SnO_2_ nanowires is ∼7.5 MΩ, which is greater than that of the SnO_2_-core/ZnO-shell nanostructures (∼3.5 MΩ). The thickness of the shell is also another important parameter in gas sensing [32]. In gas sensors, the thickness of the shell material must be close to the Debye length of nanomaterials for the sensor to exhibit enhanced sensitivity [31]. This effect is called a synergistic effect. The value of the Debye length for metal oxides is in the range of 3–30 nm, which depends on the sensing material, charge carrier concentration and the ambient temperature [61,62]. Our result shows that the shell thickness of the nanostructures grown for 30 min is ∼20 nm (see Figure 4b). Therefore, SnO_2_-core/ZnO-shell nanowires grown for 30 min exhibit enhanced ethanol sensing compared to just SnO_2_ nanowire, because of the mechanisms explained above.

As described above, the proposed mechanism for the enhanced ethanol sensing of the SnO_2_-core/ZnO-shell hierarchical nanostructures grown for 60 min and 90 min is attributed to the synergistic effect of the encapsulation of SnO_2_ by ZnO nanostructures and the increased surface sites in the hierarchical structure. On the other hand, if the thickness of the shell is larger than 45 nm, the core/shell nanostructures have weak sensing characteristics [32]. In our previous work [41], we reported that the thickness of the ZnO shell increased to ∼50 nm when the core/shell nanostructures were grown for a longer time of 120 min. Thus, this sensor could not meet the synergistic effect, due to the increased shell thickness of 50 nm. Moreover, the thickness of the ZnO shell is larger compared to other core-shell nanostructures, due to the added amount of Zn condensed on the nanostructures with increased growth time. Therefore, SnO_2_-core/ZnO-shell nanostructures grown for 120 min behave just like ZnO nanowire sensors. For a longer growth time of 120 min, the branches of the SnO_2_-core/ZnO-shell hierarchical nanostructures tend to merge into coarser structures (see Figure 3e). As a result, sensing surface areas are reduced compared to other hierarchical core/shell nanostructures. Therefore, the sensor fabricated using SnO_2_-core/ZnO-shell hierarchical nanostructures deposited at 120 min exhibits lower sensing response than SnO_2_-core/ZnO-shell hierarchical nanostructures deposited at 90 min, because of their reduced sensing surface and shell thickness being greater than the Debye length. From these results, we can conclude that the size and morphology of nanostructures have significant influence on their sensing response to ethanol gas.

## 4. Conclusions

In conclusion, a highly sensitive device for ethanol gas sensing at 400 °C has been successfully developed using SnO_2_-core/ZnO-shell nanostructures by a single-step carbon-assisted thermal evaporation method. For an optimal growth time of 90 min of SnO_2_-core/ZnO-shell nanostructures exhibit high sensitivity (31.9 for 20 ppm), high selectivity (K_ethanol/methane_ reaches 32 for 20 ppm of test gas) and a fast response and recovery behavior at 400 °C. Thus, SnO_2_-core/ZnO-shell nanostructures prepared by a facile fabrication method are promising candidates for the detection of ethanol gas with desired performances. This improvement in ethanol gas response is attributed to the large surface area providing active sites for gas-solid interactions and the synergistic effect of the encapsulation of SnO_2_ by ZnO nanostructures.
